# Serotonin Control of Thermotaxis Memory Behavior in Nematode *Caenorhabditis elegans*

**DOI:** 10.1371/journal.pone.0077779

**Published:** 2013-11-01

**Authors:** Yinxia Li, Yunli Zhao, Xu Huang, Xingfeng Lin, Yuling Guo, Daoyong Wang, Chaojun Li, Dayong Wang

**Affiliations:** 1 Key Laboratory of Developmental Genes and Human Disease in Ministry of Education, Medical School of Southeast University, Nanjing, China; 2 The School of Medicine, Nanjing University, Nanjing, China; Zhejiang University School of Medicine, China

## Abstract

*Caenorhabditis elegans* is as an ideal model system for the study of mechanisms underlying learning and memory. In the present study, we employed *C. elegans* assay system of thermotaxis memory to investigate the possible role of serotonin neurotransmitter in memory control. Our data showed that both mutations of *tph-1*, *bas-1*, and *cat-4* genes, required for serotonin synthesis, and mutations of *mod-5* gene, encoding a serotonin reuptake transporter, resulted in deficits in thermotaxis memory behavior. Exogenous treatment with serotonin effectively recovered the deficits in thermotaxis memory of *tph-1* and *bas-1* mutants to the level of wild-type N2. Neuron-specific activity assay of TPH-1 suggests that serotonin might regulate the thermotaxis memory behavior by release from the ADF sensory neurons. Ablation of ADF sensory neurons by expressing a cell-death activator gene *egl-1* decreased the thermotaxis memory, whereas activation of ADF neurons by expression of a constitutively active protein kinase C homologue (*pkc-1*(*gf*)) increased the thermotaxis memory and rescued the deficits in thermotaxis memory in *tph-1* mutants. Moreover, serotonin released from the ADF sensory neurons might act through the G-protein-coupled serotonin receptors of SER-4 and SER-7 to regulate the thermotaxis memory behavior. Genetic analysis implies that serotonin might further target the insulin signaling pathway to regulate the thermotaxis memory behavior. Thus, our results suggest the possible crucial role of serotonin and ADF sensory neurons in thermotaxis memory control in *C. elegans*.

## Introduction

Memory can be defined as encoding, storage and retrieval of learning inputs in human and animals [1]. Nematode *Caenorhabditis elegans* is an excellent model organism to study the biological processes relevant to a wide variety of human behavior and the related disease systems [2]. Early in 1990, the *C. elegans* was suggested as an ideal model system for study of the mechanisms underlying learning and memory [3]. The worms have a simple nervous system comprising 302 neurons which are all well identified, and the connectivity between all neurons has been fully mapped [4]–[5]. Especially, many components such as neurotransmitters and their respective receptors in the nervous system of *C. elegans* are similar to those in mammals [4]. *C. elegans* displays several forms of memory, such as memory to thermotaxis, chemotaxis, and mechanotransduction [1].

In *C. elegans*, thermotaxis memory is one of the widely used assay system to elucidate the mechanism of memory behavior [1], [6]–[8]. For the neuronal circuit of thermotaxis memory, it has been supposed that the worms may adjust the stored set-point of thermotactic memory to their cultivation temperature at least through AFD (a sensory neuron)-AIY (an interneuron, the major postsynaptic partner of AFD)-AIZ (an interneuron, the major postsynaptic partner of AIY) neuronal circuit [1],[6]. AFD neuron can function as not only a temperature sensor but also a temperature memory device [6]. In this supposed neuronal circuit, DGK-3 in AFD [9] and NCS-1 in AIY [10] regulate the thermotaxis memory in *C. elegans*.

In *C. elegans*, the thermal information and food state can be integrated and processed in interneurons by monoamines such as serotonin to generate output behaviors [11]. *tph-1*, encoding a tryptophan hydroxylase, and *bas-1*, encoding an aromatic amino acid decarboxylase, are required for serotonin synthesis [12]–[13], and the tryptophan hydroxylase requires a cofactor whose synthesis requires a GTP cyclohydrolase I encoded by *cat-4* gene [13]. *mod-5* gene encodes a serotonin transporter, and *ser-1*, *ser-4*, *ser-7*, and *mod-1* encode serotonin receptors [14]. Nevertheless, it is unclear whether the serotonin neurotransmitter is involved in the control of thermotaxis memory behavior in worms. Thus, in the present study, we investigated the role of serotonin neurotransmitter in regulating thermotaxis memory in *C. elegans*. Our results highlight the possible crucial role of serotonin and ADF sensory neurons in regulating thermotaxis memory in *C. elegans*. Our data here will be useful for our further understanding the neuronal mechanism of neurotransmitter control of memory behavior.

## Results

### Genes Required for Serotonin Synthesis Regulate the Thermotaxis Memory Behavior in *C. elegans*

**Figure 1:**
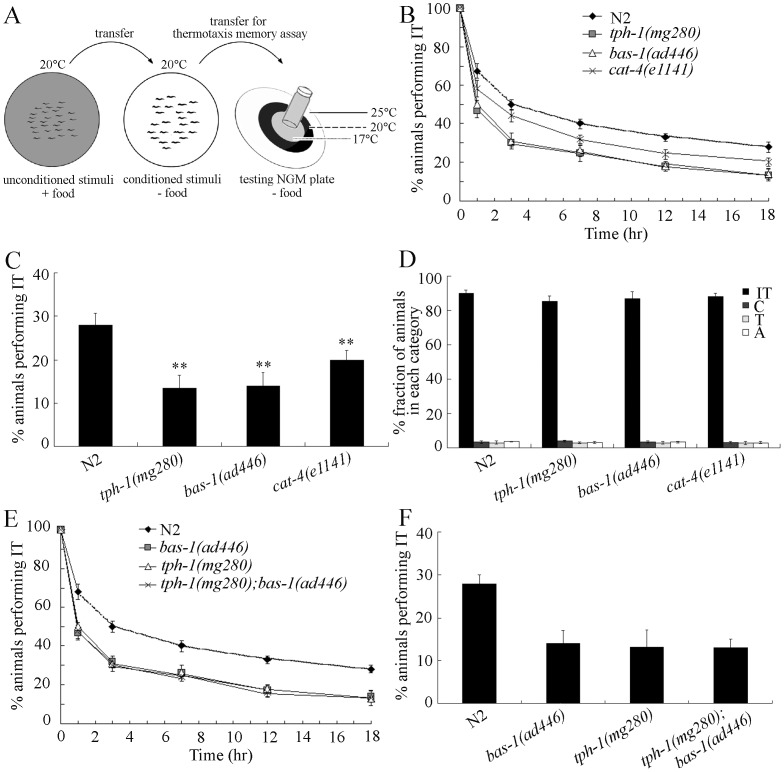
Thermotaxis memory behavior of mutants for genes required for serotonin synthesis. (A) Thermotaxis memory assay model [7]. (B) Extinction of the association (food at 20°C) of mutant animals. The normalized isothermal tracking behavior (IT) values were used. (C) Comparison of the extinctions of wild-type and mutant animals at the time interval of 18-hr. (D) Thermotaxis behavior of wild-type and mutant animals. In the thermotaxis assay system, movement to 25°C was scored as thermophilic (T); movement to 17°C was scored as cryophilic (C); movement across the thermal gradient (17°C/25°C) was scored as athermotactic (A); and movement at 20°C was scored as IT. (E–F) Genetic interaction of *tph-1* with *bas-1* in regulating thermotaxis memory. Bars represent means ± S.E.M. **p<0.01.

In *C. elegans*, mutations of *tph-1* or *bas-1* cause deficits in serotonin synthesis [12]–[13]. We first employed the thermotaxis memory assay model (Fig. 1A) to investigate the memory behaviors in *tph-1* and *bas-1* mutants. Because nematodes tend to migrate toward their cultivation temperature after conditioning (food at 20°C) and move along this isotherm on a radical temperature gradient, the assay model is used to assess a form of memory for thermosensation (Fig. 1A). In this thermotaxis memory assay model, mutations of *tph-1* and *bas-1* genes shortened the extinction period of the association paradigm (food at 20°C) for different time intervals compared with wild-type N2 nematodes (Fig. 1B). In addition, mutation of *cat-4* gene, involved in control of TPH-1 activity [13], also decreased memory behavior compared with wild-type N2 nematodes (Fig. 1B). In the assay model, the percentages of animals performing isothermal tracking behavior (IT) at the time interval of 18-hr were significantly decreased in *tph-1(mg280)*, *bas-1(ad446)*, and *cat-4(e1141)* mutants compared with wild-type N2 (Fig. 1C). Compared with the phenotype that wild-type N2 needs 3-hr to perform the half maximal extinction, *tph-1(mg280)* and *bas-1(ad446)* mutants used approximately 1-hr and *cat-4(e1141)* mutants used approximately 2-hr to finish the half maximal extinction (Fig. 1B). To ensure that the altered thermotaxis memory behaviors in *tph-1*, *bas-1*, and *cat-4* mutants were not due to the deficits in thermotaxis behavior, we examined the thermotaxis behaviors in *tph-1(mg280)*, *bas-1(ad446)*, and *cat-4(e1141)* mutants. Our data showed that *tph-1(mg280)*, *bas-1(ad446)*, and *cat-4(e1141)* mutants exhibited the similar thermotaxis phenotype to that of wild-type N2 (Fig. 1D). These data demonstrate that genes required for serotonin synthesis are essential for thermotaxis memory control in *C. elegans*. Our results also imply the possible important role of neurotransmitter of serotonin in thermotaxis memory control.

We also investigated the genetic interaction of *tph-1* and *bas-1* in regulating thermotaxis memory. As shown in Figs. 1E-1F, double mutants of *tph-1(mg280);bas-1(ad446)* exhibited the similar deficits in thermotaxis memory to that in *tph-1(mg280)* or *bas-1(ad446)* mutants, suggesting that *tph-1* and *bas-1* may function in a same genetic pathway in regulating the thermotaxis memory in *C. elegans*.

### Effects of Exogenous Serotonin Treatment on Thermotaxis Memory Behavior of *tph-1* and *bas-1* Mutants

**Figure 2:**
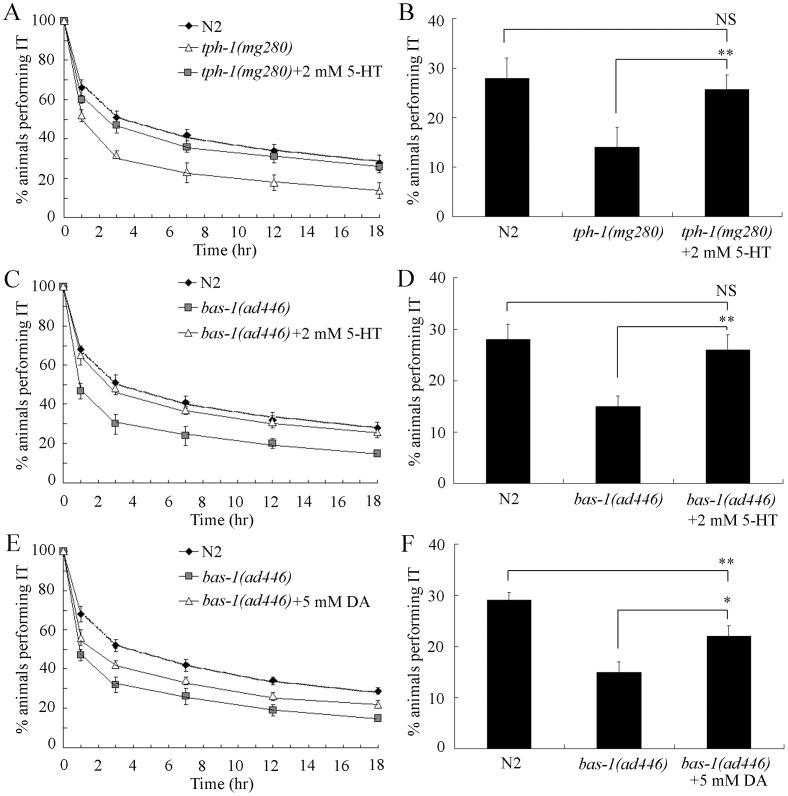
Effects of exogenous serotonin and dopamine on thermotaxis memory behavior in *tph-1* or *bas-1* mutants. (A–B) Effects of exogenous serotonin on thermotaxis memory behavior in *tph-1* mutants. (C–D) Effects of exogenous serotonin on thermotaxis memory behavior in *bas-1* mutants. (E–F) Effects of exogenous dopamine on thermotaxis memory behavior in *bas-1* mutants. Serotonin or dopamine was dissolved in M9 buffer and spread on the NGM plates at a final concentration of 2 mM or 5 mM. The normalized isothermal tracking behavior (IT) values were used. 5-HT, serotonin; DA, dopamine. Bars represent means ± S.E.M. *p<0.05, **p<0.01. NS, no significance.

To further investigate the role of serotonin in thermotaxis memory control, we examined the effects of exogenous treatment with 2 mM of serotonin on thermotaxis memory behavior in *tph-1* and *bas-1* mutants. Exogenous treatment with 2 mM of serotonin effectively recovered the deficits in thermotaxis memory of *tph-1(mg280)* and *bas-1(ad446)* mutants to the level of wild-type N2 (Figs. 2A–2D). Serotonin treated *tph-1(mg280)* and *bas-1(ad446)* mutants exhibited the similar half maximal extinction and percentages of animals performing IT at the time interval of 18-hr to those of wild-type N2 (Figs. 2A–2D).

Considering the fact that *bas-1* gene is also required for dopamine synthesis [16], we also examined the effects of exogenous treatment with 5 mM of dopamine on thermotaxis memory behavior in *bas-1* mutants. Exogenous treatment with 5 mM of dopamine only partially recovered the deficits in thermotaxis memory of *bas-1(ad446)* mutants (Figs. 2E–2F). Under our experimental conditions, dopamine treated *bas-1(ad446)* mutants still exhibited the decreased half maximal extinction and percentages of animals performing IT at the time interval of 18-hr compared with those in wild-type N2 (Figs. 2E–2F). Under our experimental conditions, exogenous treatment with 2 mM of serotonin or 5 mM of dopamine did not significantly influence the thermotaxis memory behavior in wild-type N2 nematodes (data not shown). Therefore, although we did not exclude the important role of dopamine in thermotaxis memory control, our data suggest the possible pivotal role of serotonin in regulating the thermotaxis memory in *C. elegans*.

### Genetic Interaction of TPH-1 with Insulin Signaling Pathway in Regulating Thermotaxis Memory

**Figure 3:**
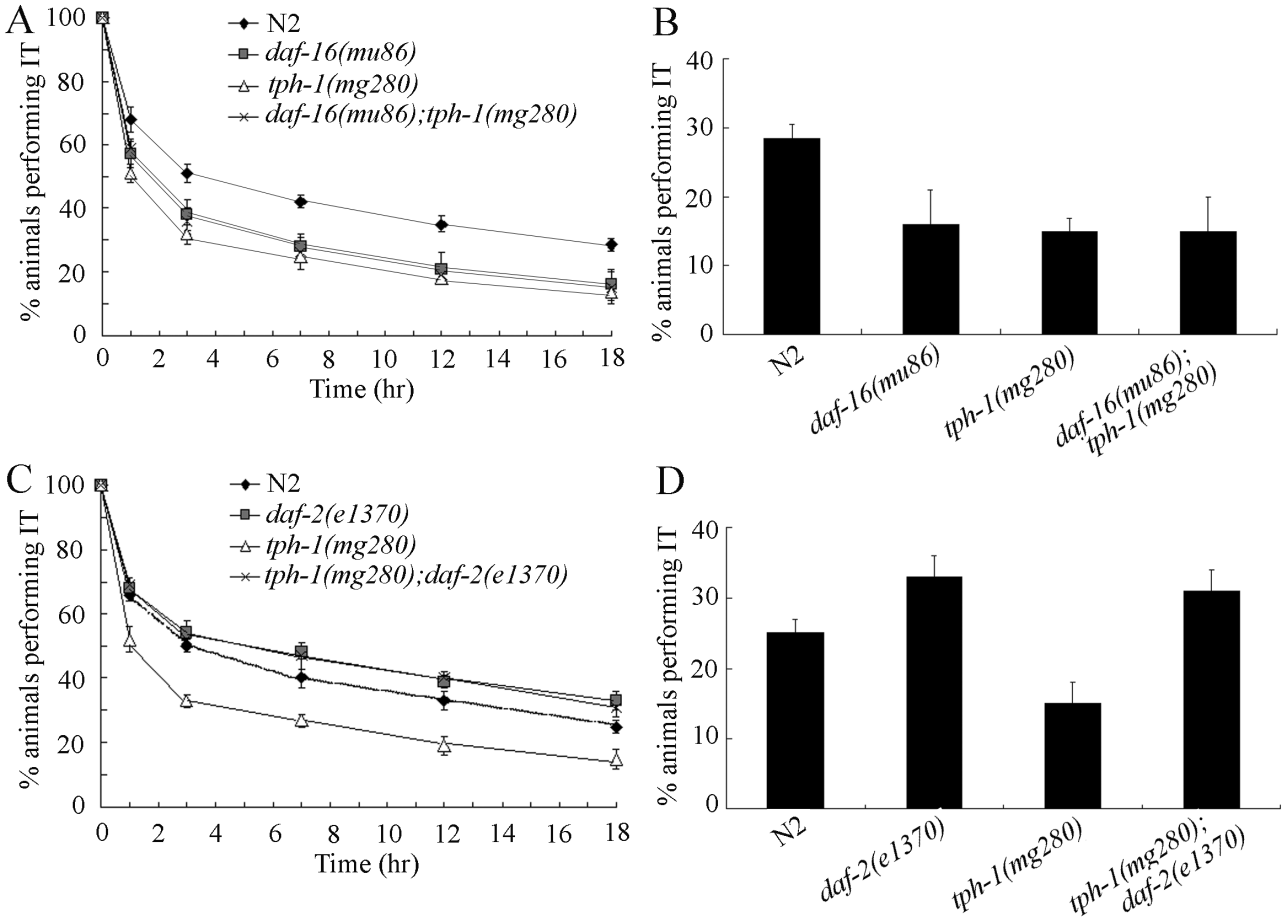
Genetic interaction of *tph-1* with insulin signals in regulating thermotaxis memory. (A–B) Genetic interaction of *tph-1* with *daf-16* in regulating thermotaxis memory. (C–D) Genetic interaction of *tph-1* with *daf-2* in regulating thermotaxis memory. The normalized isothermal tracking behavior (IT) values were used. Bars represent means ± S.E.M.

To determine the genetic mechanism of serotonin in regulating thermotaxis memory, we next investigated the genetic interaction of TPH-1 with insulin signaling pathway in regulating thermotaxis memory. Previous study demonstrated that serotonin may target the neuro-endocrine pathway from the DAF-2 insulin/IGF receptor to the DAF-16 FOXO transcription factor to modulate the response to environmental and physiological stresses [17]. Mutations of *daf-16* caused the decrease in thermotaxis memory; however, mutations of *daf-2* resulted in the increase in thermotaxis memory (Figs. 3A and 3D). *daf-16(mu86)* mutants showed the decreased half maximal extinction and percentages of animals performing IT at the time interval of 18-hr, but *daf-2(e1370)* mutants exhibited the increased half maximal extinction and percentages of animals performing IT at the time interval of 18-hr compared with those in wild-type N2 (Figs. 3A and 3D). Both *daf-16(mu86)* and *daf-2(e1370)* mutants had the normal thermotaxis behavior (Fig. S1). More interestingly, we found that double mutants of *daf-16(mu86);tph-1(mg280)* exhibited the similar thermotaxis memory to that of *daf-16(mu86)* mutants, and double mutants of *tph-1(mg280);daf-2(e1370)* showed the similar thermotaxis memory to that *daf-2(e1370)* mutants (Figs. 3A and 3D). There results imply that, serotonin may also target the insulin signaling pathway to regulate the thermotaxis memory behavior in *C. elegans*.

### Effects of Serotonin Reuptake Transporter MOD-5 on Thermotaxis Memory Behavior

**Figure 4:**
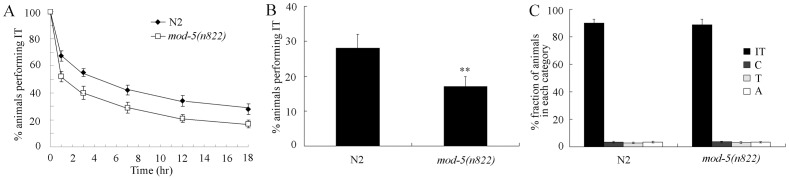
Thermotaxis memory behavior in *mod-5* mutants. (A) Extinction of the association (food at 20°C) of wild-type and *mod-5(n822)* mutant animals. The normalized isothermal tracking behavior (IT) values were used. (B) Comparison of the extinctions of wild-type and *mod-5(n822)* mutant animals at the time interval of 18-hr. (C) Thermotaxis behavior of wild-type and *mod-5(n822)* mutant animals. In the thermotaxis assay system, movement to 25°C was scored as thermophilic (T); movement to 17°C was scored as cryophilic (C); movement across the thermal gradient (17°C/25°C) was scored as athermotactic (A); and movement at 20°C was scored as IT. Bars represent means ± S.E.M. **p<0.01.

Moreover, we found that mutations of *mod-5* gene, encoding a serotonin reuptake transporter, also significantly decreased the thermotaxis memory compared with wild-type N2 (Fig. 4A). *mod-5(n822)* mutants exhibited the decreased half maximal extinction and percentages of animals performing IT at the time interval of 18-hr (Figs. 4A and 4B). *mod-5(n822)* mutants showed the normal thermotaxis behavior like wild-type N2 (Fig. 4C).

### Neuronal Circuit of Serotonin in Regulating Thermotaxis Memory Behavior

**Figure 5:**
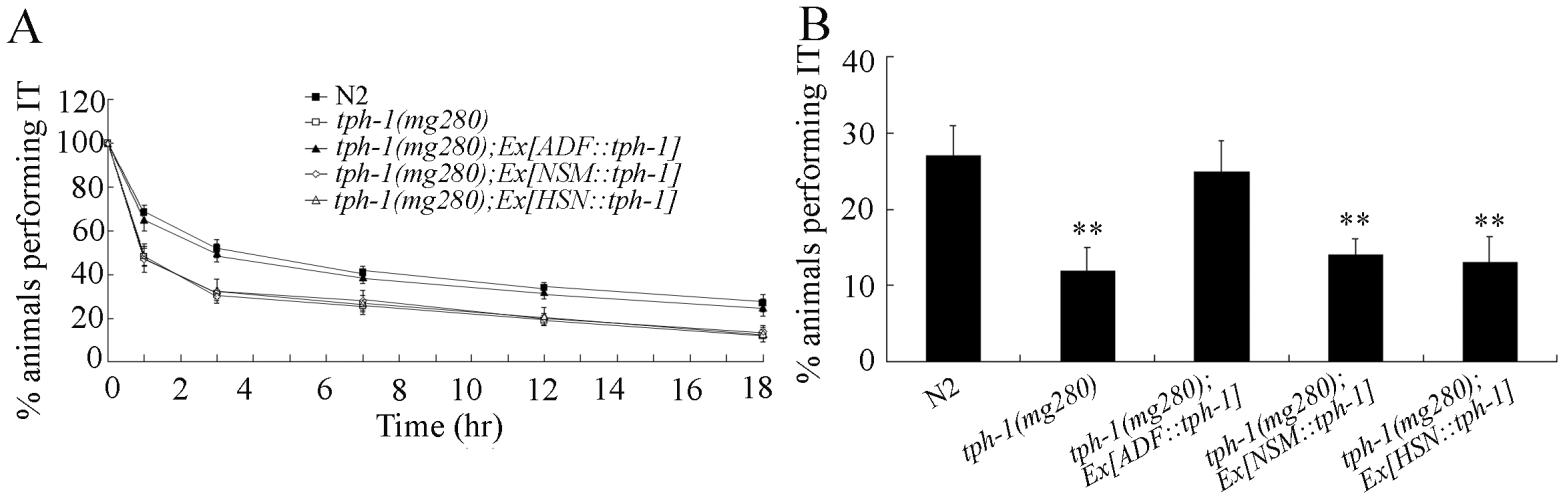
Neuron-specific activities of TPH-1 in regulating thermotaxis memory behavior. (A) Effects of TPH-1 expression in different neurons on extinction of the association (food at 20°C) in *tph-1(mg280)* mutants. (B) Effects of TPH-1 expression in different neurons on extinctions of animals at the time interval of 18-hr in *tph-1(mg280)* mutants. The normalized isothermal tracking behavior (IT) values were used. Bars represent means ± S.E.M. **p<0.01.

In *C. elegans*, *tph-1* gene expression is limited to only a few serotonergic neurons such as ADF, NSM, HSN [12], [15], [18]. We further investigated the neuron-specific activities of TPH-1 in regulating thermotaxis memory behavior in *C. elegans*. Neuron-specific expression of TPH-1 in ADF sensory neurons rescued the deficits in thermotaxis memory of *tph-1(mg280)* mutants; however, expression of TPH-1 in NSM and HSN neurons did not significantly influence the phenotype of thermotaxis memory behavior in *tph-1(mg280)* mutants (Fig. 5, Table S1). *tph-1(mg280)* mutants expressing *tph-1* gene in ADF sensory neurons exhibited the similar half maximal extinction and percentages of animals performing IT at the time interval of 18-hr to those in wild-type N2 (Fig. 5, Table S1). These data suggest that serotonin may regulate the thermotaxis memory behavior through release from the ADF sensory neurons in *C. elegans*.

### Role of ADF Sensory Neurons in Thermotaxis Memory Control

**Figure 6:**
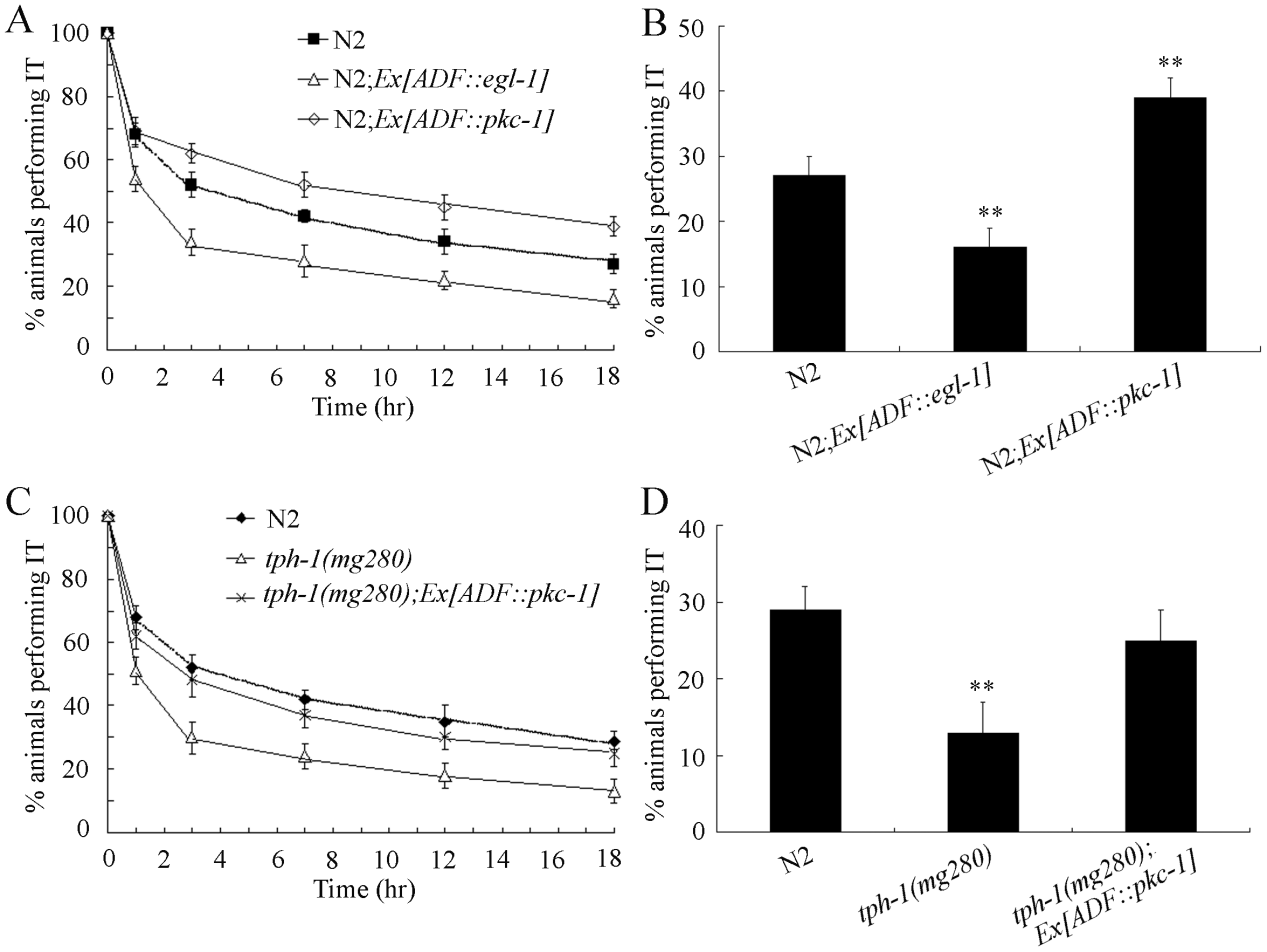
Activation and genetically ablation of ADF sensory neurons affect the thermotaxis memory behavior in *C. elegans*. (A–B) Effects of activation of ADF sensory neurons by expressing *pkc-1* gene and genetically ablation of ADF sensory neurons by expressing a cell-death activator gene *egl-1* on thermotaxis memory behavior. (C–D) Effects of activation of ADF sensory neurons by expressing *pkc-1* gene on thermotaxis memory behavior in *tph-1(mg280)* mutants. The normalized isothermal tracking behavior (IT) values were used. Bars represent means ± S.E.M. **p<0.01.

To determine the possible important role of ADF sensory neurons in regulating thermotaxis memory, we constructed the transgenic strains with activation or genetic ablation of ADF sensory neurons in *C. elegans*. We examined the thermotaxis memory behavior in a strain in which ADF sensory neurons were genetically ablated by expressing the cell-death activator gene *egl-1* [20] under control of *srh-142* promoter, and found that nematodes lacking ADF sensory neurons exhibited a decrease in the thermotaxis memory (Figs. 6A and 6B; Table S2). In contrast, activation of ADF sensory neurons by expression of a constitutively active protein kinase C homologue of *C. elegans* (*pkc-1*(*gf*)) that can promotes synaptic transmission and neuropeptide release [21] resulted in a increase in the thermotaxis memory (Figs. 6A and 6B; Table S2). Animals lacking ADF sensory neurons exhibited a decrease in both the half maximal extinction and the percentages of animals performing IT at the time interval of 18-hr, whereas animals with activation of ADF sensory neurons showed an increase in both the half maximal extinction and the percentages of animals performing IT at the time interval of 18-hr (Figs. 6A and 6B; Table S2). Nematodes lacking ADF sensory neurons or with activation of ADF sensory neurons showed the normal thermotaxis behavior compared with wild-type N2 (Fig. S2). More interestingly, we observed that expression of *pkc-1* in ADF sensory neurons could rescue the deficits in thermotaxis memory of *tph-1(mg280)* mutants (Figs. 6C and 6D; Table S2). These results imply the crucial role of ADF sensory neurons for proper thermotaxis memory behavior in *C. elegans*.

Because the *tph-1(mg280)* mutant has no detectable serotonin [12], and PKC-1 was suggested to be able to regulate the secretion of neuropeptides [21], we further examined whether *pkc-1* gene was doing something else other than promoting secretion of serotonin in nematodes. INS-1, FLP-6, and NLP-3 are neuropeptides expressing in ADF sensory neurons [22]–[24]. We found that expression of INS-1 and NLP-3 in ADF sensory neurons could effectively rescue the deficits in thermotaxis memory of *tph-1(mg280)* mutants; however, expression of FLP-6 in ADF sensory neurons did not rescue the deficits in thermotaxis memory of *tph-1(mg280)* mutants (Fig. S3; ; Table S3). These results suggest that some other neuropeptides such as INS-1 and NLP-3 can be co-released from the ADF sensory neurons and contribute the regulation of memory behavior in *tph-1* mutants.

### G-protein-coupled Serotonin Receptors SER-4 and SER-7 Regulate the Thermotaxis Memory Behavior

**Figure 7:**
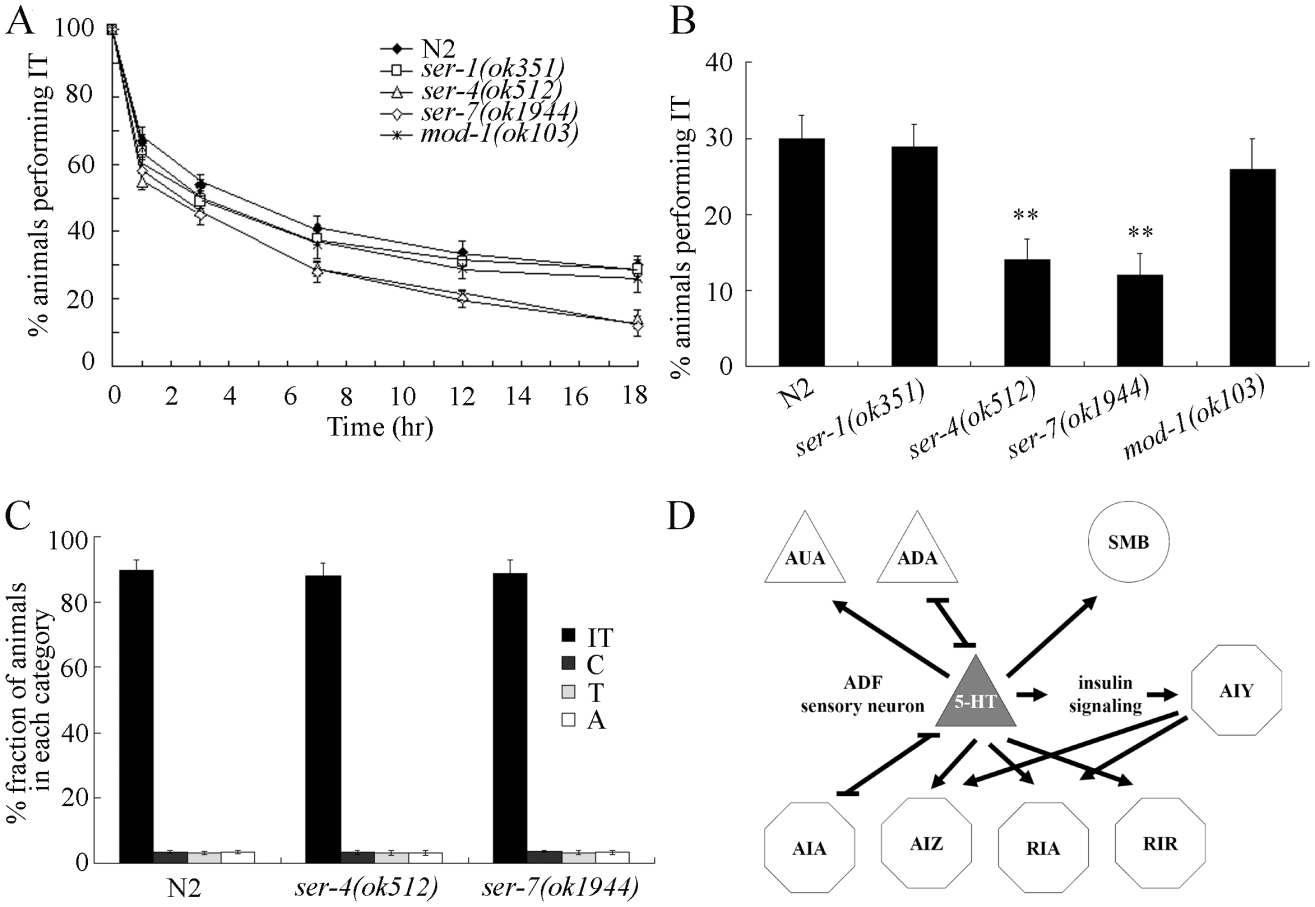
Effects of mutations of serotonin receptor genes on thermotaxis memory behavior. (A) Extinction of the association (food at 20°C) of wild-type and serotonin receptor mutants. The normalized isothermal tracking behavior (IT) values were used. (B) Comparison of the extinctions of wild-type and serotonin receptor mutants at the time interval of 18-hr. (C) Thermotaxis behavior of wild-type and serotonin receptor mutants. In the thermotaxis assay system, movement to 25°C was scored as thermophilic (T); movement to 17°C was scored as cryophilic (C); movement across the thermal gradient (17°C/25°C) was scored as athermotactic (A); and movement at 20°C was scored as IT. (D) One model for the neuronal regulation of thermotaxis memory behavior by serotonin signal. Serotonin may be released from ADF sensory neurons to regulate the thermotaxis memory behavior through the functions of its receptors of SER-4 and SER-7 in the still unknown neurons. Serotonin may also regulate the activity of AIY interneurons by targeting insulin signaling pathway, and then AIY interneurons further regulate thermotaxis memory through modulating the activity of AIZ and/or RIA interneurons. Sensory neurons are shown as triangles, interneurons as hexagons, and motor neurons as circuits. Bars represent means ± S.E.M. **p<0.01.

In *C. elegans*, *ser-1*, *ser-4*, and *ser-7* genes encode the G-protein-coupled serotonin receptors, and *mod-1* gene encodes the ionotropic serotonin receptor [14]. To further examine the molecular control of serotonin signal on thermotaxis memory, we investigated the thermotaxis memory behavior in serotonin receptor mutants. Compared with wild-type N2, *ser-1(ok351)* and *mod-1(ok103)* mutants did not exhibit the obvious deficits in thermotaxis memory behavior (Figs. 7A and 7B). In contrast, *ser-4(ok512)* and *ser-7(ok1944)* mutants showed the significantly decreased thermotaxis memory behavior compared with wild-type N2 (Figs. 7A and 7B). *ser-4(ok512)* and *ser-7(ok1944)* mutants exhibited the decrease in both the half maximal extinction and the percentages of animals performing IT at the time interval of 18-hr (Figs. 7A and 7B). Meanwhile, *ser-4(ok512)* and *ser-7(ok1944)* mutants had the normal thermotaxis behavior compared with wildtype N2 (Fig. 7C). In *C. elegans*, the *ser-4(ok512);ser-7(ok1944)* double mutants showed the similar thermotaxis memory behavior to that in *ser-4(ok512)* or *ser-7(ok1944)* single mutants (Fig. S4). Therefore, serotonin released from ADF sensory neurons may further act through its receptors SER-4 and SER-7 in still unknown neurons to regulate the thermotaxis memory behavior in *C. elegans* (Fig. 7D).

## Discussion

Serotonin is one of the important neurotransmitters for animals and human beings, and has been linked to emotional and motivational aspects of human behavior [22]. Several of previous clinical studies have implied the possible important role of serotonin in learning and memory behavior [25]. Nevertheless, whether the role of serotonin is related to memory behavior of human still remains an important question [25]. In the present study, we provide the evidence to prove the crucial role of serotonin in memory control with the aid of thermotaxis memory assay model in *C. elegans*. Firstly, mutations of genes (*tph-1*, and *bas-1*) encoding enzymes required for serotonin synthesis [12]–[13] caused the significant decrease in thermotaxis memory behavior (Fig. 1), which suggests that serotonin not only regulates pharyngeal pumping and egg-laying [12] but also regulates learning [17] and memory in *C. elegans*. Secondly, exogenous treatment with serotonin could rescue the deficits in thermotaxis memory of *tph-1(mg280)* and *bas-1(ad446)* mutants (Fig. 2). Serotonin is also critical for rewarded olfactory short-term memory in Drosophila, another invertebrate model animal [26]. Besides the serotonin, our data further imply the possible involvement of dopamine in thermotaxis memory control (Figs. 2E and 2F), although dopamine may have no the same contribution to thermotaxis memory as serotonin. The dopamine synthesis may help modulate default activity during working memory in younger adults [27].

*mod-5(n822)* mutants were hypersensitive to exogenous serotonin, and hyper-responsive in the experience-dependent enhanced slowing response to food modulated by serotonin [19]. Our data further demonstrate that mutations of *mod-5* gene resulted in deficits in thermotaxis memory behavior in *C. elegans* (Fig. 4). Therefore, deficits in both synthesis and uptake of serotonin will noticeably influence the thermotaxis memory behavior in *C. elegans*. Previous studies have implied that the serotonin transporter expression seems to be a reliable neuronal marker related to memory mechanisms, its alterations and potential treatment [28]. In addition, it was reported that human serotonin transporter polyadenylation polymorphism can modulate the retention of fear extinction memory [29].

Our data demonstrate that serotonin regulate the thermotaxis memory through its receptors of SER-4 and SER-7, because, among the examined serotonin receptor mutants, only *ser-4(ok512)* and *ser-7(ok1944)* mutants exhibited deficits in thermotaxis memory behavior (Fig. 7). SER-4 and SER-7 are G-protein-coupled serotonin receptors [14]. In *C. elegans*, both ionotropic serotonin receptor and G-protein-coupled serotonin receptors are involved in the control of stress response [17]. Ionotropic serotonin receptor MOD-1 also regulates olfactory learning behavior [30]. These data imply that *C. elegans* can employ different types of serotonin receptors to regulate different behaviors during the development. Moreover, considering that fact that deficit in memory is an important phenotype of many disorders, the further study on serotonin control of memory in *C. elegans* may provide some useful clues for clinical assessment or analysis of contribution of serotonin to the occurrence and regulation of the related disorders.

For the molecular control of thermotaxis memory behavior by serotonin, we hypothesized here that serotonin may target insulin signaling pathway to modulate the thermotaxis memory behavior. Genetic analysis indicates that double mutants of *daf-16(mu86);tph-1(mg280)* showed the similar thermotaxis memory phenotype to that of *daf-16(mu86)*, and double mutants of *tph-1(mg280);daf-2(e1370)* showed the similar thermotaxis memory phenotype to that of *daf-2(e1370)* (Fig. 3). Previous study further suggests that G-protein-coupled serotonin receptor SER-4 modulated the starvation-induced DAF-16 nuclear accumulation [17]. In *C. elegans*, serotonin and insulin signaling pathway may function together to regulate the integration of thermal information and food state in interneurons such as AIY and AIZ [6], [11].

The very simple neuronal structure makes *C. elegans* an ideal model animal to examine the neuronal circuit of a specific behavior [31]. The assay of neuron-specific activity of TPH-1 indicates that TPH-1 functioned in ADF sensory neurons to regulate the thermotaxis memory behavior (Fig. 5; Table S1). In *C. elegans*, sensory inputs can be released from ADF sensory neurons to the AUA sensory neurons, AIZ, RIA, and RIR interneuons, and SMB motor neurons, and ADF sensory neurons can further form gap junctions with ADA sensory neurons and AIA interneurons (Fig. 7D). *ser-4* gene expresses in PVT, RIB, RIS, and other unidentified head neurons, and *ser-7* gene may at least express in M4 motor neuron and several pharyngeal neurons [14]. Therefore, so far we still did not know in which neurons serotonin released from ADF sensory neurons may activate the corresponding serotonin receptors of SER-4 and SER-7 to modulate the thermotaxis memory behavior.

Previous studies imply the possible involvement of AFD sensory neurons and AIY interneurons in thermotaxis memory control [9]–[10]. In the present study, we further provide the evidence to prove the crucial role of ADF sensory neurons in thermotaxis memory control. Ablation of ADF sensory neurons decreased the thermotaxis memory behavior, whereas activation of ADF sensory neurons increased the thermotaxis memory behavior (Fig. 6; Table S2). In *C. elegans*, sensory inputs can be released from AIY interneurons to the AIZ and RIA interneurons [31]. Thus, for the serotonin control of thermotaxis memory behavior, one possibility is that serotonin may function through SER-4 and SER-7 in unknown neurons. The another possibility is that serotonin may regulate the activity of AIY interneurons by targeting insulin signaling pathway, and then AIY interneurons may further regulate the thermotaxis memory behavior through modulating the activity of AIZ and/or RIA interneurons (Fig. 7D). Nevertheless, because ablation of AFD, AIY, AIZ, and RIA neurons will cause deficits in thermotaxis behavior [32], so far the direct evidence for AFD, AIY, AIZ, and RIA neurons in regulating thermotaxis memory behavior is still largely absent.

In conclusion, our data demonstrate that serotonin was required for the thermotaxis memory control in *C. elegans*. Deficits in both synthesis and transport of serotonin influenced the thermotaxis memory behavior. Serotonin released from ADF sensory neurons acted on G-protein-coupled serotonin receptors SER-4 and SER-7 in still unknown neurons to regulate the thermotaxis memory behavior. ADF sensory neurons may function together with AFD and AIY neurons to modulate the thermotaxis memory behavior. The further identification of neuronal circuit for serotonin receptors of SER-4 and SER-7 to regulate thermotaxis memory behavior is necessary. Moreover, it is still unclear whether the serotonin control of memory is a relatively conserved mechanism for all the assay systems of memory behavior in *C. elegans* [1]–[4], [6], [30], [33].

## Materials and Methods

### Strains and Genetics

Wild-type nematodes were *C. elegans* variety Bristol, strain N2. Nematodes were grown on nematode growth medium (NGM) plates seeded with *Escherichia coli* OP50 at 20°C as described [34]. The following strains were used in the current study: wild-type N2, *tph-1(mg280)*, *bas-1(ad446)*, *cat-4(e1141)*, *daf-16(mu86)*, *daf-2(e1370)*, *mod-5(n822)*, *ser-1(ok351)*, *ser-4(ok512)*, *ser-7(ok1944)*, and *mod-1(ok103)*, originally obtained from the *Caenorhabditis* Genetics Center (funded by the NIH National Center for Research Resource, USA). Gravid animals were washed off the plates into centrifuge tubes and were lysed with a bleaching mixture (0.45 N NaOH, 2% HOCl). Age synchronous populations of young adults were obtained by the collection as described [35]. Double mutant strains without additional marker mutations were constructed using standard genetic methods and verified by complementation testing.

### Tracks of Animals on a Radial Thermal Gradient

The procedure for the themotaxis assay using a radial temperature gradient was performed according to previous description [32]. A radial thermal gradient was created on an agar surface in the 9-cm Petri dish, in which a steeper gradient, ranging from approximate 17°C at the central area to approximate 25°C at the periphery, was formed [36]. A radial gradient of temperature was created by placing a vial containing frozen acetic acid on the bottom of the plate and incubating the plate at 26°C for 90-min in the presence of a constant humidity of 60%. Nematodes were raised in the presence of food at 20°C on a 6 cm petri dish filled with NGM containing 1.7% agar, 0.25% bacto peptone, 50 mM NaCl, 25 mM potassium phosphate, pH 6.0, and then transferred onto a fresh plate devoid of bacteria for 2-min. Individual nematodes were deposited on a 9-cm Petri dish with a thermal gradient, and allowed to move freely for 2-hr. Upon removal of nematode from the plates, tracks left on the agar surface were recorded. Movement to 25°C was scored as thermophilic (T); movement to 17°C was scored as cryophilic (C); movement across the thermal gradient (17°C/25°C) was scored as athermotactic (A); movement at 20°C was scored as isothermal tracking behavior (IT) [37]. In the thermotaxis assay system, the radial temperature gradient from 17 to 26°C was measured as described previously [36]–[37]. Five replicates were performed each assay.

### Thermotaxis Memory Assay

The thermotaxis memory assay was performed as previously described [7], . As shown in Fig. 1A, a cohort of synchronized young adult animals was incubated on NGM plates with fresh food (unconditioned stimuli) at 20°C overnight, and then washed twice with M9 buffer and transferred to an unseeded plate (conditioned stimuli) at 20°C for different incubation intervals (0, 1, 3, 7, 12, and 18-hr). Individual nematodes (30 worms) were deposited on a 9-cm Petri dish with a thermal gradient, and allowed to move freely for 2-hr. Percentages of animals performing IT behavior at 20°C were determined by the tracks left on agar surface upon removal of animals from the plates. Five replicates were performed each assay.

### Pharmacological Experiments

Serotonin was dissolved in M9 buffer and spread on the NGM plates at a final concentration of 2 mM, and dopamine was dissolved in M9 buffer and spread on the NGM plates at a final concentration of 5 mM. Young adults were treated on these NGM plates for 2-hr [15], and then transferred to NGM plates containing the examined drug with conditioned stimuli for thermotaxis memory assay. Three replicates were performed each assay.

### Molecular Biology Methods

To generate entry vectors carrying promoter sequences, the promoter regions were amplified by PCR from *C. elegans* genomic DNA (1.8 kb for *srh-142* promoter used for ADF-specific expression, 3.7 kb for *ceh-2* promoter used for NSM-specific expression, and 3.3 kb for *unc-86* promoter used for expression in HSN). And then these promoters were inserted into the pPD95_77 vector in the sense orientation. *tph-1*, *pkc-1*, *egl-1, ins-1, flp-6,* and *nlp-3* cDNAs were amplified by polymerase chain reaction (PCR). The sequences of the amplified cDNA were verified by sequencing, and then the cDNA was inserted into corresponding entry vectors carrying promoter sequence. Germline transformation was performed as described [38] by coinjecting the testing DNA at a concentration of 10–40 µg/mL and the marker DNA of P*dop-1::rfp* at a concentration of 60 µg/mL into the gonad of nematodes.

### Statistical Analysis

All data in this article were expressed as means ± standard error of the mean (S.E.M.). Graphs were generated using Microsoft Excel (Microsoft Corp., Redmond, WA). Statistical analysis was performed using SPSS 12.0 (SPSS Inc., Chicago, USA). Differences between groups were determined using analysis of variance (ANOVA). The probability levels of 0.05 and 0.01 were considered statistically significant.

## Supporting Information

Figure S1 **Thermotaxis behavior of wild-type and** ***daf-2*** **and** ***daf-16*** **mutants.** In the thermotaxis assay system, movement to 25°C was scored as thermophilic (T); movement to 17°C was scored as cryophilic (C); movement across the thermal gradient (17°C/25°C) was scored as athermotactic (A); and movement at 20°C was scored as isothermal tracking behavior (IT). Bars represent means ± S.E.M.
(DOC)

Figure S2 **Thermotaxis behavior of animals lacking ADF sensory neurons or with activation of ADF sensory neurons.** In the thermotaxis assay system, movement to 25°C was scored as thermophilic (T); movement to 17°C was scored as cryophilic (C); movement across the thermal gradient (17°C/25°C) was scored as athermotactic (A); and movement at 20°C was scored as isothermal tracking behavior (IT). Bars represent means ± S.E.M.
(DOC)

Figure S3 **Rescue of deficits in thermotaxis memory in** ***tph-1*** **mutants by expressing neuropeptides in ADF sensory neurons.** (A) Extinction of the association (food at 20°C) of wild-type, *tph-1* mutant, and *tph-1* mutant animals expressing neuropeptides in ADF sensory neurons. The normalized isothermal tracking behavior (IT) values were used. (B) Comparison of the extinctions of wild-type, *tph-1* mutant, and *tph-1* mutant animals expressing neuropeptides in ADF sensory neurons at the time interval of 18-hr. Bars represent means ± S.E.M. **p<0.01.
(DOC)

Figure S4 **Genetic interaction of SER-4 with SER-7 in regulating thermotaxis memory.** (A) Extinction of the association (food at 20°C) of wild-type and mutant animals. The normalized isothermal tracking behavior (IT) values were used. (B) Comparison of the extinctions of wild-type and mutant animals at the time interval of 18-hr. Bars represent means ± S.E.M.
(DOC)

Table S1 **Effects of TPH-1 expression in different neurons on phenotype of thermotaxis memory in *tph-1* mutants.**
(DOC)

Table S2 **Effects of activation and genetically ablating of ADF sensory neurons on thermotaxis memory behavior.**
(DOC)

Table S3 **Thermotaxis memory in wild-type, *tph-1* mutant, and *tph-1* mutant animals expressing neuropeptides in ADF sensory neurons.**
(DOC)

## References

[1] Ye H-Y, Ye B-P, Wang D-Y (2008) Molecular control of memory in nematode *Caenorhabditis elegans* . Neurosci Bull 24: 49–55. 18273077 10.1007/s12264-008-0808-9 PMC5552524

[2] Ardiel EL, Rankin CH (2010) An elegant mind: learning and memory in *Caenorhabditis elegans* . Learn Mem 17: 191–201. 20335372 10.1101/lm.960510

[3] Rankin CH, Beck CD, Chiba CM (1990) *Caenorhabditis elegans*: a new model system for the study of learning and memory. Behav Brain Res 37: 89–92. 2310497 10.1016/0166-4328(90)90074-o

[4] Giles AC, Rankin CH (2009) Behavioral and genetic characterization of habituation using *Caenorhabditis elegans* . Neurobiol Learn Mem 92: 139–146. 18771741 10.1016/j.nlm.2008.08.004

[5] Hu Y-O, Sun Y, Ye B-P, Wang D-Y (2007) Computational analysis of genetic loci required for amphid structure and functions and their possibly corresponding microRNAs in *C. elegans* . Neurosci Bull 23: 9–20. 17592520 10.1007/s12264-007-0002-5 PMC5500771

[6] Sasakura H, Mori I (2013) Behavioral plasticity, learning, and memory in *C. elegans* . Curr Opin Neurobiol 23: 92–99. 23063296 10.1016/j.conb.2012.09.005

[7] Ye H-Y, Ye B-P, Wang D-Y (2008) Trace administration of vitamin E can retrieve and prevent UV-irradiation- and metal exposure-induced memory deficits in nematode *Caenorhabditis elegans* . Neurobiol Learn Mem 90: 10–18. 18191592 10.1016/j.nlm.2007.12.001

[8] Ye H-Y, Ye B-P, Wang D-Y (2008) Evaluation of the long-term memory for the thermosensation regulation by NCS-1 in *Caenorhabditis elegans* . Neurosci Bull 24: 1–6. 18273069 10.1007/s12264-008-0920-x PMC5552526

[9] Biron D, Shibuya M, Gabel C, Wasserman SM, Clark DA, et al (2006) A diacylglycerol kinase modulates long-term thermotactic behavioral plasticity in *C. elegans* . Nat Neurosci 9: 1499–1505. 17086178 10.1038/nn1796

[10] Gomez M, de Castro E, Guarin E, Sasakura H, Kuhara A, et al (2001) Ca^2+^ signaling via the neuronal calcium sensor-1 regulates associative learning and memory in *C. elegans* . Neuron 30: 241–248. 11343658 10.1016/s0896-6273(01)00276-8

[11] Mori I, Sasakura H, Kuhara A (2007) Worm thermotaxis: a model system for analyzing thermosensation and neural plasticity. Curr Opin Neurobiol 17: 712–719. 18242074 10.1016/j.conb.2007.11.010

[12] Sze JY, Victor M, Loer C, Shi Y, Ruvkun G (2000) Food and metabolic signaling defects in a *Caenorhabditis elegans* serotonin-synthesis mutant. Nature 403: 560–564. 10676966 10.1038/35000609

[13] Loer CM, Kenyon CJ (1993) Serotonin-deficient mutants and male mating behavior in the nematode *Caenorhabditis elegans* . J Neurosci 13: 5407–5417. 8254383 10.1523/JNEUROSCI.13-12-05407.1993 PMC6576401

[14] Chase DL, Koelle MR (2007) Biogenic amine neurotransmitters in *C. elegans* Wormbook, ed. The *C. elegans* Research Community, doi: 10.1895/wormbook.1.132.1. 10.1895/wormbook.1.132.1 PMC4781333 18050501

[15] Li Z, Li Y, Yi Y, Huang W, Yang S, et al. (2012) Dissecting a central flip-flop circuit that integrates contradictory sensory cues in *C. elegans* feeding regulation. Nat Commun doi: 10.1038/ncomms1780. 10.1038/ncomms1780 22491324

[16] Hare EE, Loer CM (2004) Function and evolution of the serotonin-synthesis *bas-1* gene and other aromatic amino acid decarboxylase genes in *Caenorhabditis elegans* . BMC Evol Biol 4: 24. 15287963 10.1186/1471-2148-4-24 PMC514703

[17] Liang B, Moussaif M, Kuan C, Gargus JJ, Sze JY (2006) Serotonin targets the DAF-16/FOXO signaling pathway to modulate stress responses. Cell Metab 4: 429–440. 17141627 10.1016/j.cmet.2006.11.004

[18] Loer CM, Davidson B, McKerrow JA (1999) A Phenylalanine hydroxylase gene from the nematode *C. elegans* is expressed in the hypodermis. J Neurogenet 13: 157–180. 10928216 10.3109/01677069909083472

[19] Ranganathan R, Sawin ER, Trent C, Horvitz HR (2001) Mutations in the *Caenorhabditis elegans* serotonin reuptake transporter MOD-5 reveal serotonin-dependent and –independent activities of fluoxetine. J Neurosci 21: 5871–5884. 11487610 10.1523/JNEUROSCI.21-16-05871.2001 PMC6763176

[20] Styer K, Singh V, Macosko E, Steele SE, Bargmann CI, et al (2008) Innate immunity in *Caenorhabditis elegans* is regulated by neurons expressing NPR-1/GPCR. Science 322: 460–464. 18801967 10.1126/science.1163673 PMC2831475

[21] Sieburth D, Madison JM, Kaplan JM (2007) PKC-1 regulates secretion of neuropeptides. Nat Neurosci 10: 49–57. 17128266 10.1038/nn1810

[22] Tomioka M, Adachi T, Suzuki H, Kunitomo H, Schafer WR, et al (2006) The insulin/PI 3-kinase pathway regulates salt chemotaxis learning in *Caenorhabditis elegans* . Neuron 51: 613–625. 16950159 10.1016/j.neuron.2006.07.024

[23] Chang S, Johnston RJ, Hobert O (2003) A transcriptional regulatory cascade that controls left/right asymmetry in chemosensory neurons of *C. elegans* . Genes Dev 17: 2123–2137. 12952888 10.1101/gad.1117903 PMC196454

[24] Nathoo AN, Moeller RA, Westlund BA, Hart AC (2001) Identification of neuropeptide-like protein gene families in *Caenorhabditis elegans* and other species. Proc Natl Acad Sci USA 98: 14000–14005. 11717458 10.1073/pnas.241231298 PMC61156

[25] Meneses A, Liy-Salmeron G (2012) Serotonin and emotion, learning and memory. Rev Neurosci 23: 543–553. 23104855 10.1515/revneuro-2012-0060

[26] Sitaraman D, LaFerriere H, Birman S, Zars T (2012) Serotonin is critical for rewarded olfactory short-term memory in *Drosophila* . J Neurogenet 26: 238–244. 22436011 10.3109/01677063.2012.666298

[27] Braskie MN, Landau SM, Wilcox CE, Taylor SD, O’Neil JP, et al (2011) Correlations of striatal dopamine synthesis with default network deactivations during working memory in younger adults. Hum Brain Mapp 32: 947–961. 20578173 10.1002/hbm.21081 PMC3176660

[28] Meneses A, Perez-Garcia G, Ponce-Lopez T, Tellez R, Castillo C (2011) Serotonin transporter and memory. Neuropharmacology 61: 355–363. 21276807 10.1016/j.neuropharm.2011.01.018

[29] Hartley CA, McKenna MC, Salman R, Holmes A, Casey BJ, et al (2012) Serotonin transporter polyadenylation polymorphism modulates the retention of dear extinction memory. Proc Natl Acad Sci USA 109: 5493–5498. 22431634 10.1073/pnas.1202044109 PMC3325655

[30] Zhang Y, Lu H, Bargmann CI (2005) Pathogenic bacteria induce aversive olfactory learning in *Caenorhabditis elegans* . Nature 438: 179–184. 16281027 10.1038/nature04216

[31] White JG, Southgate E, Thomson JN, Brenner S (1986) The structure of the nervous system of the nematode *Caenorhabditis elegans* . Philos Tran Royal Soc B Biol Sci 314: 1–340. 10.1098/rstb.1986.0056 22462104

[32] Mori I, Ohshima Y (1995) Neural regulation of thermotaxis in *Caenorhabditis elegans* . Nature 376: 344–348. 7630402 10.1038/376344a0

[33] Wang W-H, Cheng L-C, Pan F-Y, Xue B, Wang D-Y, et al (2011) Intracellular trafficking of histone deacetylase 4 regulates long-term memory formation. Anat Rec 294: 1025–1034. 10.1002/ar.21389 21542139

[34] Brenner S (1974) The genetics of *Caenorhabditis elegans* . Genetics 77: 71–94. 4366476 10.1093/genetics/77.1.71 PMC1213120

[35] Donkin SG, Williams PL (1995) Influence of developmental stage, salts and food presence on various end points using *Caenorhabditis elegans* for aquatic toxicity testing. Environ Toxicol Chem 14: 2139–2147.

[36] Mori I, Ohshima Y (1997) Molecular neurogenetics of chemotaxis and thermotaxis in the nematode *Caenorhabditis elegans.* . BioEssays 19: 1055–1064. 9454556 10.1002/bies.950191204

[37] Mohri A, Kodama E, Kimura KD, Koike M, Mizuno T, et al (2005) Genetic control of temperature preference in the nematode *Caenorhabditis elegans* . Genetics 169: 1437–1450. 15654086 10.1534/genetics.104.036111 PMC1449549

[38] Mello C, Fire A (1995) DNA transformation. Methods Cell Biol 48: 451–482. 8531738

