# Supplementary material for: Serotonin Control of Thermotaxis Memory Behavior in Nematode Caenorhabditis elegans
Source: PLoS One. 2013 Nov 1;8(11):e77779. doi: 10.1371/journal.pone.0077779 (PMC3815336; doi:10.1371/journal.pone.0077779)
Supplement: Figure S2 — Thermotaxis behavior of animals lacking ADF sensory neurons or with activation of ADF sensory neurons. In the thermotaxis assay system, movement to 25°C was scored as thermophilic (T); movement to 17°C was scored as cryophilic (C); movement across the thermal gradient (17°C/25°C) was scored as athermotactic (A); and movement at 20°C was scored as isothermal tracking behavior (IT). Bars represent means ± S.E.M. (DOC) [file pone.0077779.s002.doc]

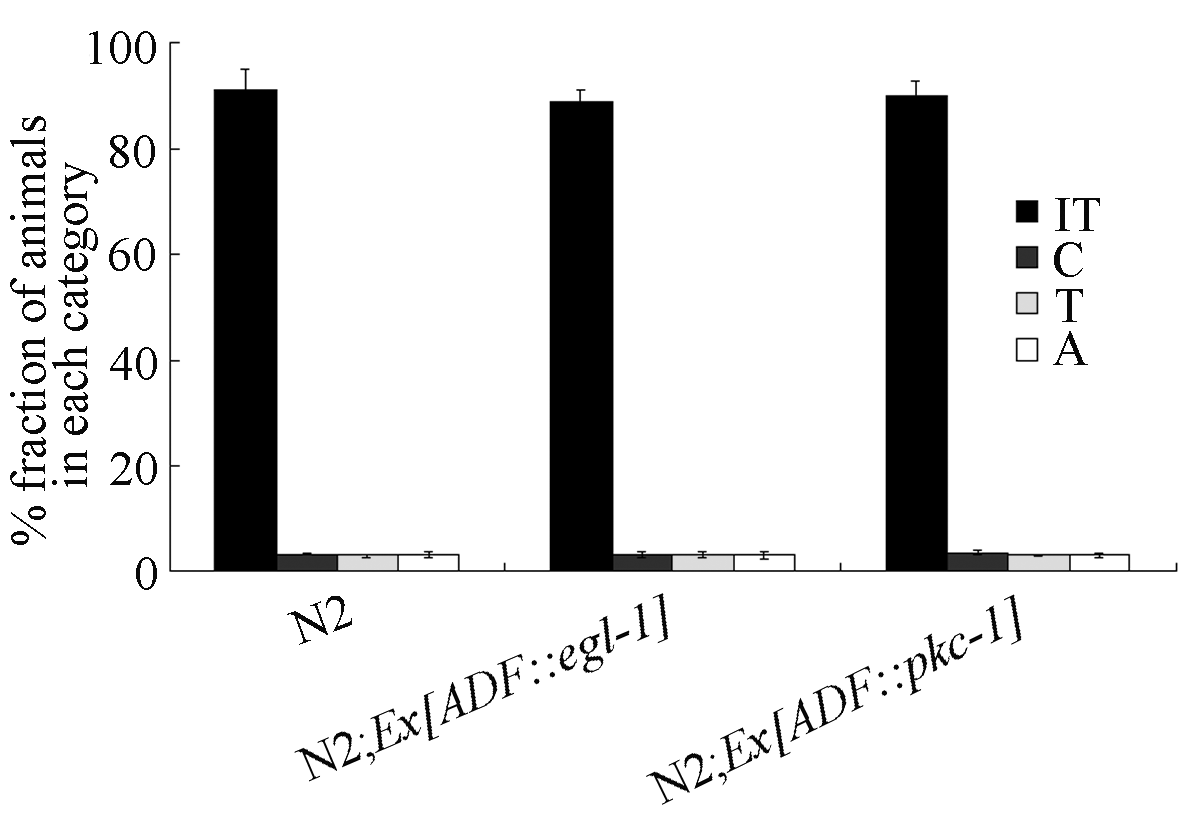


**Figure S2. Thermotaxis behavior of animals lacking ADF sensory neurons or with activation of ADF sensory neurons.**  In the thermotaxis assay system, movement to 25°C was scored as thermophilic (T); movement to 17°C was scored as cryophilic (C); movement across the thermal gradient (17°C/25°C) was scored as athermotactic (A); and movement at 20°C was scored as isothermal tracking behavior (IT). Bars represent means ± S.E.M.
