# Supplementary material for: Serotonin Control of Thermotaxis Memory Behavior in Nematode Caenorhabditis elegans
Source: PLoS One. 2013 Nov 1;8(11):e77779. doi: 10.1371/journal.pone.0077779 (PMC3815336; doi:10.1371/journal.pone.0077779)
Supplement: Figure S3 — Rescue of deficits in thermotaxis memory in tph-1 mutants by expressing neuropeptides in ADF sensory neurons. (A) Extinction of the association (food at 20°C) of wild-type, tph-1 mutant, and tph-1 mutant animals expressing neuropeptides in ADF sensory neurons. The normalized isothermal tracking behavior (IT) values were used. (B) Comparison of the extinctions of wild-type, tph-1 mutant, and tph-1 mutant animals expressing neuropeptides in ADF sensory neurons at the time interval of 18-hr. Bars represent means ± S.E.M. **p<0.01. (DOC) [file pone.0077779.s003.doc]

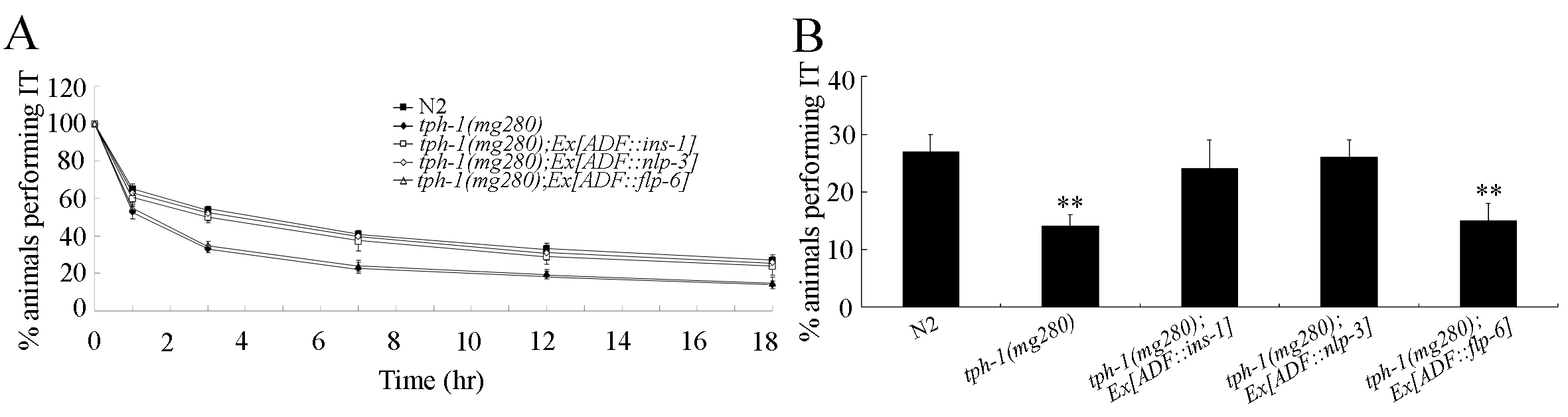


**Figure S3. Rescue of deficits in thermotaxis memory in *tph-1* mutants by expressing neuropeptides in ADF sensory neurons.**  (A) Extinction of the association (food at 20°C) of wild-type, *tph-1* mutant, and *tph-1* mutant animals expressing neuropeptides in ADF sensory neurons. The normalized isothermal tracking behavior (IT) values were used. (B) Comparison of the extinctions of wild-type, *tph-1* mutant, and *tph-1* mutant animals expressing neuropeptides in ADF sensory neurons at the time interval of 18-hr. Bars represent means ± S.E.M. ^**^*p* < 0.01.
