# Supplementary material for: Serotonin Control of Thermotaxis Memory Behavior in Nematode Caenorhabditis elegans
Source: PLoS One. 2013 Nov 1;8(11):e77779. doi: 10.1371/journal.pone.0077779 (PMC3815336; doi:10.1371/journal.pone.0077779)
Supplement: Figure S4 — Genetic interaction of SER-4 with SER-7 in regulating thermotaxis memory. (A) Extinction of the association (food at 20°C) of wild-type and mutant animals. The normalized isothermal tracking behavior (IT) values were used. (B) Comparison of the extinctions of wild-type and mutant animals at the time interval of 18-hr. Bars represent means ± S.E.M. (DOC) [file pone.0077779.s004.doc]

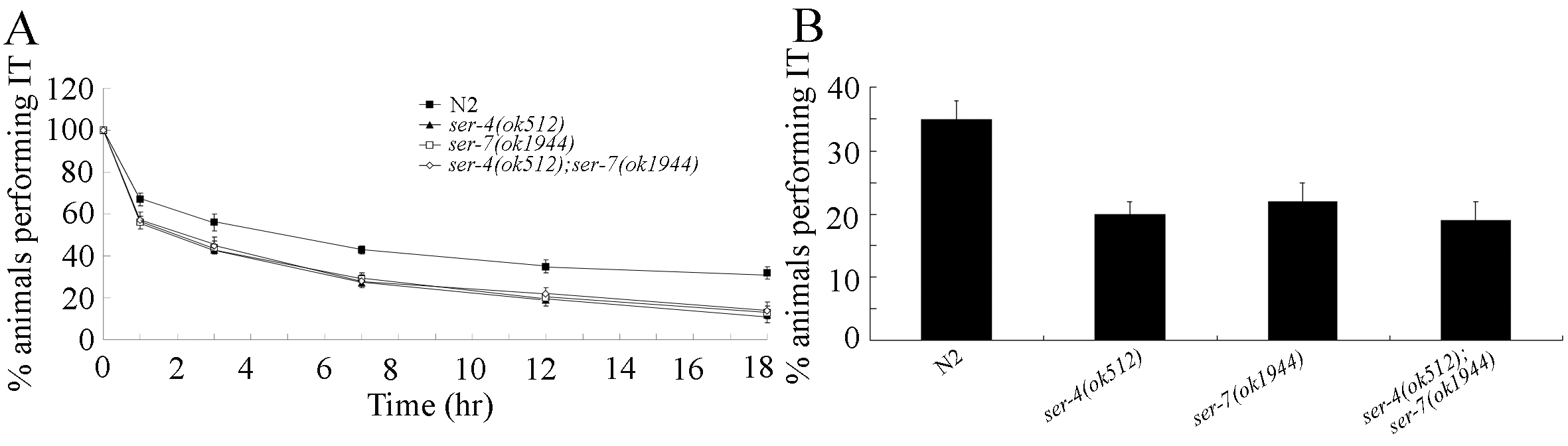


**Figure S4. Genetic interaction of SER-4 with SER-7 in regulating thermotaxis memory.**  (A) Extinction of the association (food at 20°C) of wild-type and mutant animals. The normalized isothermal tracking behavior (IT) values were used. (B) Comparison of the extinctions of wild-type and mutant animals at the time interval of 18-hr. Bars represent means ± S.E.M.
