# Supplementary material for: Serotonin Control of Thermotaxis Memory Behavior in Nematode Caenorhabditis elegans
Source: PLoS One. 2013 Nov 1;8(11):e77779. doi: 10.1371/journal.pone.0077779 (PMC3815336; doi:10.1371/journal.pone.0077779)
Supplement: Table S1 — Effects of TPH-1 expression in different neurons on phenotype of thermotaxis memory in tph-1 mutants. (DOC) [file pone.0077779.s005.doc]

**Table S1. Effects of TPH-1 expression in different neurons on phenotype of thermotaxis memory in *tph-1* mutants**

| Strain | Observed animals (n) | Percentages of animals performing IT at the time interval of 18-hr | Significance (compared with WT) |
| --- | --- | --- | --- |
| WT | 30 | 27 ± 4 |  |
| *tph-1(mg280)* | 30 | 12 ± 3 | *p* < 0.01 |
| *tph-1(mg280);Ex[ADF::tph-1]#1* | 30 | 25 ± 4 | NS |
| *tph-1(mg280);Ex[ADF::tph-1]#2* | 30 | 26 ± 3 | NS |
| *tph-1(mg280);Ex[NSM::tph-1]#1* | 30 | 14 ± 2.1 | *p* < 0.01 |
| *tph-1(mg280);Ex[NSM::tph-1]#2* | 30 | 13.5 ± 2 | *p* < 0.01 |
| *tph-1(mg280);Ex[HSN::tph-1]#1* | 30 | 13 ± 3.5 | *p* < 0.01 |
| *tph-1(mg280);Ex[HSN::tph-1]#2* | 30 | 13.2 ± 3 | *p* < 0.01 |

IT, isothermal tracking behavior. NS, no significance.
