# Supplementary material for: Serotonin Control of Thermotaxis Memory Behavior in Nematode Caenorhabditis elegans
Source: PLoS One. 2013 Nov 1;8(11):e77779. doi: 10.1371/journal.pone.0077779 (PMC3815336; doi:10.1371/journal.pone.0077779)
Supplement: Table S2 — Effects of activation and genetically ablating of ADF sensory neurons on thermotaxis memory behavior. (DOC) [file pone.0077779.s006.doc]

**Table S2. Effects of activation and genetically ablating of ADF sensory neurons on thermotaxis memory behavior**

| Strain | Observed animals (n) | Percentages of animals performing IT at the time interval of 18-hr | Significance (compared with WT) |
| --- | --- | --- | --- |
| WT | 30 | 27 ± 3 |  |
| *tph-1(mg280)* | 30 | 13 ± 4 | *p* < 0.01 |
| *N2;Ex[ADF::egl-1]#1* | 30 | 16 ±3 | *p* < 0.01 |
| *N2;Ex[ADF::egl-1]#2* | 30 | 16.4 ± 4 | *p* < 0.01 |
| *N2;Ex[ADF::pkc-1]#1* | 30 | 39 ± 3 | *p* < 0.01 |
| *N2;Ex[ADF::pkc-1]#2* | 30 | 38 ± 4 | *p* < 0.01 |
| *tph-1(mg280);Ex[ADF::pkc-1]#1* | 30 | 25 ± 4 | NS |
| *tph-1(mg280);Ex[ADF::pkc-1]#2* | 30 | 26 ± 3 | NS |

IT, isothermal tracking behavior. NS, no significance.
