# Supplementary material for: Serotonin Control of Thermotaxis Memory Behavior in Nematode Caenorhabditis elegans
Source: PLoS One. 2013 Nov 1;8(11):e77779. doi: 10.1371/journal.pone.0077779 (PMC3815336; doi:10.1371/journal.pone.0077779)
Supplement: Table S3 — Thermotaxis memory in wild-type, tph-1 mutant, and tph-1 mutant animals expressing neuropeptides in ADF sensory neurons. (DOC) [file pone.0077779.s007.doc]

**Table S3. Thermotaxis memory in wild-type, *tph-1* mutant, and *tph-1*** mutant animals expressing neuropeptides in ADF sensory neurons

| Strain | Observed animals (n) | Percentages of animals performing IT at the time interval of 18-hr | Significance (compared with WT) |
| --- | --- | --- | --- |
| WT | 30 | 27 ± 3 |  |
| *tph-1(mg280)* | 30 | 14 ± 2 | *p* < 0.01 |
| *tph-1(mg280);Ex[ADF::ins-1]#1* | 30 | 24 ± 5 | NS |
| *tph-1(mg280);Ex[ADF::ins-1]#2* | 30 | 25 ± 4 | NS |
| *tph-1(mg280);Ex[ADF::nlp-3]#1* | 30 | 26 ± 3 | NS |
| *tph-1(mg280);Ex[ADF::nlp-3]#2* | 30 | 26.5 ± 3 | NS |
| *tph-1(mg280);Ex[ADF::flp-6]#1* | 30 | 15 ± 3 | *p* < 0.01 |
| *tph-1(mg280);Ex[ADF::flp-6]#2* | 30 | 16 ± 2 | *p* < 0.01 |

IT, isothermal tracking behavior. NS, no significance.
